# Complaint-Directed Mini-Interventions for Depressive Complaints: A Randomized Controlled Trial of Unguided Web-Based Self-Help Interventions

**DOI:** 10.2196/jmir.6581

**Published:** 2017-01-04

**Authors:** Suzanne Lokman, Stephanie S Leone, Marion Sommers-Spijkerman, Agnes van der Poel, Filip Smit, Brigitte Boon

**Affiliations:** ^1^ Department of Public Mental Health Trimbos Institute: Netherlands Institute of Mental Health and Addiction Utrecht Netherlands; ^2^ Centre for eHealth and Well-being Research Department of Psychology, Health and Technology University of Twente Enschede Netherlands; ^3^ Department of Clinical, Neuro and Developmental Psychology VU University Amsterdam Netherlands; ^4^ EMGO+ Institute for Health and Care Research Department of Epidemiology and Biostatistics VU University Medical Centre Amsterdam Netherlands

**Keywords:** prevention, depression, Internet-based intervention, randomized controlled trial

## Abstract

**Background:**

Prevention of depression is important due to the substantial burden of disease associated with it. To this end, we developed a novel, brief, and low-threshold Web-based self-help approach for depressive complaints called complaint-directed mini-interventions (CDMIs). These CDMIs focus on highly prevalent complaints that are demonstrably associated with depression and have a substantial economic impact: stress, sleep problems, and worry.

**Objective:**

The aim was to evaluate the effectiveness of the Web-based self-help CDMIs in a sample of adults with mild-to-moderate depressive symptoms compared to a wait-list control group.

**Methods:**

A two-armed randomized controlled trial was conducted. An open recruitment strategy was used. Participants were randomized to either the Web-based CDMIs or the no-intervention wait-list control group. The CDMIs are online, unguided, self-help interventions, largely based on cognitive behavioral techniques, which consist of 3 to 4 modules with up to 6 exercises per module. Participants are free to choose between the modules and exercises. Assessments, using self-report questionnaires, took place at baseline and at 3 and 6 months after baseline. The control group was given access to the intervention following the 3-month assessment. The primary goal of the CDMIs is to reduce depressive complaints. The primary outcome of the study was a reduction in depressive complaints as measured by the Inventory of Depressive Symptomatology Self-Report (IDS-SR). Secondary outcomes included reductions in stress, worry, sleep problems, and anxiety complaints, and improvements in well-being. Data were analyzed using linear mixed models.

**Results:**

In total, 329 participants enrolled in the trial, of which 165 were randomized to the intervention group and 164 to the control group. Approximately three-quarters of the intervention group actually created an account. Of these participants, 91.3% (116/127) logged into their chosen CDMI at least once during the 3-month intervention period (median 3, range 0-166). After 3 months, there was a significant reduction in depressive symptomatology for participants in the intervention group compared to participants in the wait-list control group (reduction in depression: mean –4.47, 95% CI –6.54 to –2.40; Cohen *d*=–0.70). Furthermore, significant effects were observed for sleep problems, worry, anxiety, and well-being, with effect sizes ranging from –0.29 to –0.40. The intervention did not significantly reduce stress. At 6-month follow-up, the improvements in the intervention group were generally sustained.

**Conclusions:**

This study shows that the online self-help CDMIs have a positive impact on various mental health outcomes. Future research should focus on which specific strategies may boost adherence, and increase the reach of the CDMIs among people with low socioeconomic status.

**ClinicalTrial:**

Netherlands Trial Register (NTR): NTR4612; http://www.trialregister.nl/trialreg/admin/rctview.asp?TC=4612 (Archived by WebCite at http://www.webcitation.org/6n4PVYddM)

## Introduction

Depressive disorders are highly prevalent and are estimated to affect approximately 350 million people worldwide [1-3]. Depression is among the leading causes of disease burden globally [4]. Depression is also associated with substantial societal costs, with depression-related costs in Europe estimated at €92 billion annually [5].

Currently available effective pharmacological and psychological treatments only moderately reduce the enormous burden of disease associated with depression [6,7]. Therefore, interest in approaches to prevent depressive disorders has been growing. Meta-analytic studies indicate that preventive interventions can be effective in reducing the incidence rate of depressive disorders [8-10]. Moreover, depression prevention appears to be cost-effective and may even be cost-saving from a societal perspective [11,12].

However, the reach of preventive mental health interventions is far from optimal, particularly in the population with low socioeconomic status (SES), among whom the incidence and prevalence rates of depression are especially high [13-15]. In the Netherlands, for example, the lifetime prevalence of depression in the low SES population is 21.4% compared to 15.2% in the population with high SES [16]. There is a need for preventive interventions that can easily be implemented with limited costs and are suitable for a broad range of target populations. This has led to the development of the complaint-directed mini-interventions (CDMIs). These brief and low-threshold interventions focus on highly prevalent complaints that are demonstrably associated with depression, have a substantial economic impact, and are also frequently presented to the general practitioner (GP): psychological stress, sleep problems, and worry [17-21].

In line with recent symptom-network and transdiagnostic approaches to mental disorders [22-24], the unique feature of the CDMIs as an approach for depression is that they were developed by taking into account that symptoms preceding or underlying depression may not be disorder-specific (eg, worry) and may also vary by individual. Therefore, they are oriented toward complaints (symptoms) rather than disorders, thus allowing each individual to choose the complaint(s) they want to focus on.

In addition, CDMI participants are also free to choose which modules and exercises they want to do and in what order, rather than having to adhere to a fixed set of modules that are offered in a sequential manner. This latter feature of the CDMIs, which allows users to choose, has been employed in other online interventions [25,26]. Possible advantages are more user satisfaction and insight into which individual modules are of most use to users of the intervention.

The CDMIs were initially developed as group interventions and preliminary findings of a pilot study with a single-group pre-post design showed reductions in symptoms of depression, sleep, stress, and worry [27]. Importantly, interest in the CDMIs was high as evidenced by the rapid recruitment of participants into the pilot study. These findings prompted the development of an unguided Web-based self-help version of the CDMIs, with the potential to reach a large number of people at low cost. Previous findings show that online self-help interventions for depression and depressive symptoms can be effective [28]. Although guided interventions generally show larger effect sizes and adherence rates than unguided interventions [29-31], we expected the preference-based and low-threshold nature of the CDMIs to be a novel and potentially effective approach for depressive symptoms.

The primary aim of this trial was to evaluate the effectiveness of the Web-based unguided self-help CDMIs in a sample of adults with mild-to-moderate depressive symptomatology compared to a wait-list control group. We hypothesized a greater reduction in depressive complaints for the participants using the online CDMIs. A secondary aim was to evaluate the effects of the CDMIs on stress, worry, sleep, anxiety, and well-being.

## Methods

### Design

A two-armed randomized controlled trial (RCT) was conducted that compared the effectiveness of the Web-based CDMIs with a no-intervention wait-listed control group that had unlimited access to usual care. Participants were assessed at three time points: at baseline and at 3- and 6-month follow-ups. The trial is reported in accordance with the CONSORT-EHEALTH checklist V1.6.1 (see Multimedia Appendix 1). The Medical Ethics Committee of the University Medical Center Utrecht approved the study protocol in 2014, and the trial was registered at the Netherlands Trial Register on May 27, 2014 (No: NTR4612).

### Eligibility Criteria

Participants were eligible for participation if they were adults (≥18 years), had access to a computer with an Internet connection, sufficient proficiency of the Dutch language, adequate computer skills to participate in the training, and mild-to-moderate depressive symptoms defined as a score of 14 to 38 on the Inventory of Depressive Symptomatology Self-Report (IDS-SR) [32,33]. These IDS-SR cut-off scores imply that the CDMIs in this study were used for, at least but not limited to, an indicated preventive purpose. Suicidal thoughts or plans, as measured with item 18 of the IDS-SR, were a reason for exclusion (a score of >1 was used; initially a score of >0 was used, but this was deemed too strict). If participants indicated they had suicidal thoughts or plans, they were advised to contact their GP or an anonymous online platform for people with suicidal thoughts or behaviors (113Online [34]).

### Recruitment and Procedures

Participants were recruited from June 2014 to January 2015 via open recruitment (ie, through relevant websites, messages on social media, and messages in digital newsletters of the Trimbos Institute). People interested in participation were referred to a special study website where they were given more information about the study and could register to take part in the study by completing a written or an online informed consent form including their name and email address. Once informed consent was given, the eligibility criteria were assessed. Applicants were requested to complete the first part of the self-report online baseline questionnaire, which acted as a screening instrument and consisted of the IDS-SR and questions about age, Internet access, and computer skills. People who did not fulfill the inclusion criteria were informed during or immediately after completing the first part of the baseline questionnaire and the reason for exclusion was provided.

Eligible participants received the second part of the online baseline questionnaire. To be randomized, the applicants were required to complete the entire baseline questionnaire. To be able to conduct the stratified block randomization, applicants were asked which CDMI they would want to take part in (“sleep better,” “stress less,” or “worry less”). Randomization to the intervention group or control group occurred automatically using a 1:1 ratio. Directly after randomization, participants allocated to the experimental condition received an email with an activation code for creating an online account that gave them access to the CDMI of their choice. The account was valid for one year. Participants in the control condition were sent an email with the outcome of the randomization, including the message that the CDMI would become accessible to them after 3 months.

### Intervention

The CDMIs are Web-based self-help interventions without therapist guidance [35], developed by the Trimbos Institute, a nonprofit organization. There are three different CDMIs: “sleep better,” “stress less,” and “worry less.” The CDMIs target people with mild-to-moderate symptoms of depression who experience problems with sleep, stress, or worry. In the interventions, users learn to better understand and deal with the problem of their choice. In fact, a unique feature of the CDMIs as an approach for depressive symptoms is that they were developed taking into account that symptoms of depression or a developing depression vary by individual. Therefore, they are complaint-focused rather than disorder-focused, thus participants can choose the CDMI they want to use based on their personal needs and do not have to use CDMIs that are not relevant to their situation. The primary goal of the CDMIs is to reduce or prevent depressive complaints. Secondary goals are to reduce sleep problems, stress, or worry.

The content of the CDMIs is largely based on cognitive behavioral techniques and incorporates elements from solution-focused therapy, mindfulness, and positive psychology. The CDMIs are made up of three to four modules, each module consisting of four to six exercises. Some modules are relevant for sleep, stress, and worry and are, therefore, part of all three CDMIs (eg, the module “relaxation”). Fixed elements in every CDMI are a home page, a diary, a list with the user’s favorite exercises, an exercise book, a to-do list, and a library. A more detailed account of the CDMIs (including screenshots) can be found in Multimedia Appendix 2. To gain access to the CDMIs, an account had to be created by entering a username and email address. Users were free to choose between the modules and exercises and could work independently through the CDMI, without supervision. The advised amount of time to spend on the CDMI was 2 to 3 hours a week for a period of at least 4 weeks.

In case any assistance was needed during the study, a contact form on the website of the CDMIs could be used or participants in the study could email or call one of the researchers. Participants were allowed to use any other type of care in addition to the online CDMIs.

### Control Group

Participants in the control group were placed on a waiting list and received access to the online CDMI of their choice after 3 months (following the 3-month postbaseline assessment). The wait-listed participants were aware of this procedure. Participants in the control group were also free to use any other types of care.

### Outcomes

#### Measurements

Participants received an email with a personal link to the online questionnaires at baseline (T0) and at 3 and 6 months after baseline (T1 and T2). At every assessment, up to three reminder emails were sent and a reminder phone call was made in case participants did not complete the survey. If there was an indication of suicidal thoughts or plans during the measurement at T1 or T2, these participants were given the same recommendation as at baseline, namely to contact their GP or an anonymous online platform for people with suicidal thoughts or behaviors (113Online [34]). After completion of the questionnaire at T1, the participants in the control condition gained access to the CDMIs provided that they had no suicidal thoughts or plans and no severe depressive symptoms (ie, score >39 on the IDS-SR) as measured at the 3-month postbaseline assessment. Six months after baseline there was an additional assessment (T2) to ascertain whether the effects of the CDMIs were sustained in the experimental condition and to obtain a postintervention measurement in the control condition. The primary and secondary outcomes were assessed at each measurement point.

#### Primary Outcome

The primary outcome of the study was depressive symptomatology as measured by the IDS-SR [32,33]. The IDS-SR consists of 30 items relating to the last 7 days that cover nine diagnostic symptom domains used to characterize a major depressive episode as well as items to define melancholic and atypical symptom features, commonly associated symptoms (eg, irritability, anxiety), and features of endogenous symptoms defined by the Research Diagnostic Criteria [36]. Items are scored on a four-point Likert scale and can be summed to obtain a total score. Scores range from zero to 84, with higher scores indicating greater depressive symptom severity.

#### Secondary Outcomes

Secondary outcomes consisted of the complaints targeted by the CDMIs and additional psychological outcomes, namely sleep problems, stress, worry, anxiety symptoms, and well-being.

To determine the frequency of sleep problems, the Jenkins Sleep Evaluation Questionnaire (JSEQ) was used [37].This questionnaire assesses the frequency of sleep problems in the past month and has been shown to have good internal consistency [27,37]. It consists of four items that are rated on a six-point scale (0=not at all; 5=22-31 days). To obtain a total score, the items are summed. Hence, the range of the total score is zero to 20, with higher scores representing greater sleep disturbance.

Stress was evaluated with the Perceived Stress Scale (PSS-10) [38]. The PSS consists of 10 questions and measures the degree to which situations in one’s life are appraised as stressful. People are asked to rate on a scale of zero to four (0=never; 4=very often) how often they experienced specific feelings and thoughts during the last month. Scores are summed to produce a total score ranging from zero to 40, with higher scores indicating more stress. Internal consistency has been found to be good (Cronbach alpha range .78-.90) [27,39].

The Penn State Worry Questionnaire (PSWQ) was used to assess the intensity, excessiveness, and uncontrollability of worry [40]. This version consists of 11 items. Respondents can indicate to what degree each item applies to them by giving a score on a five-point Likert scale (1=not at all typical of me; 5=very typical of me). A total score is computed by summing all items (range 11-55), with higher scores indicating a stronger tendency to worry. This instrument shows good psychometric properties including Cronbach alpha as high as .93 [40-43].

For assessing the severity of anxiety symptoms, the Generalized Anxiety Disorder Scale (GAD-7) was used [44,45]. Respondents are asked to rate seven items over the last 2 weeks on a four-point scale (0=not at all sure; 4=nearly every day). The sum of the seven items represents the total score (range 0-28), with higher scores defining a higher level of anxiety severity. Internal validity is good (Cronbach alpha range .88-.92) [27,45].

Well-being was measured with the Warwick-Edinburgh Mental Well-being Scale (WEMWBS) [46]. All 14 items of this scale are positively worded, cover topics of positive mental health, and show good reliability [27,47]. Items are scored on a five-point Likert scale and summed to obtain a total score, which ranges from 14 to 70. Higher scores indicate a higher state of well-being.

#### Covariates and Illness Characteristics

Demographic variables were assessed at baseline. Data on age (in years), gender (male/female), living arrangements (single/living together), educational level (low/high), work status (paid/unpaid), and gross wage (more/less than €2618, the average monthly Dutch income for 2014) were obtained. Duration of complaints was assessed with an item using a five-point scale asking “how long have the complaints been present?” and dichotomized into less than 1 year (0=a few weeks; 1=a few months; 2=6 months to a year) or 1 year or longer (3=approximately one year; 4=more than one year). Severity of complaints was assessed by an item asking participants to rate their experienced symptom severity level on a five-point scale. Severity of complaints was merged into two categories: low severity (0=not or 1=rather severe) and high severity (2=severe, 3=more than severe, or 4=very severe).

#### Use of and Satisfaction With Intervention

At the 3-month assessment (T1), the participants in the intervention group were asked to rate 15 statements covering satisfaction with the content, effects, usefulness, and overall satisfaction with the CDMIs. Suggestions for improving the intervention were also elicited. Items were rated on a five-point scale (1=completely disagree; 5=completely agree). An example of an item is “The intervention helped me deal with my complaints.” Overall satisfaction with the intervention was rated on a scale one to 10 (1=not at all satisfied; 10=very satisfied). Furthermore, participants were asked to estimate the number of minutes that they spent, on average, using the intervention during the 3-month intervention period. For the control group, the satisfaction and use questions were incorporated in the 6-month assessment because they gained access to the CDMI following the T1 assessment. In addition, user logs were used to determine the number of log-ins per participant.

#### Sample Size

Based on Lipsey and Wilson [48], we aimed to detect a standardized effect size (Cohen *d*) of at least 0.33, which corresponds to the lower bound of a medium effect size. To achieve this effect size of 0.33 with a power of 0.80 and a two-tailed test with alpha of .05, a sample size of 146 participants per condition was needed, thus 292 participants in total. The sample size was calculated prior to the start of the study using Stata version 12.1.

#### Randomization

To guarantee an even distribution of participants with different complaints and education level across the two study conditions, stratified block randomization was used. The block randomization was conducted in a block size of six, stratified by two blocks for level of education (low, high) and three blocks for the preferred type of CDMI intervention (stress, sleep problems, or worry). A computer-generated random allocation sequence was obtained using RANDOM.ORG, which was performed and handled by an independent researcher outside of the research team.

#### Blinding

The participants could not be blinded because they needed to be informed about whether they could start immediately after randomization or after 3 months.

### Statistical Analysis

Descriptive statistics were used to describe the characteristics of the study sample at baseline. Attrition analysis was conducted by comparing demographic characteristics and primary and secondary outcome variables of the participants who completed the questionnaire at T1 with those who did not complete the T1 assessment. For this purpose, differences between the groups were tested using independent *t* tests and chi-square statistics.

Analyses of the effectiveness of the CDMIs were carried out according to the intention-to-treat principle. Linear mixed models were used to estimate the effects of the CDMIs on the primary and secondary outcomes. This technique allows for the correlation between longitudinal data and uses all available data points, thus not discarding cases due to a missing value. Missing values are accounted for using the maximum likelihood method to estimate coefficients. A random intercept was fitted with an identity covariance structure. Time was defined as the within-group factor and the study condition (CDMI or wait-list) as the between-groups factor. The mean difference in the outcomes between the study conditions over time is expressed by the condition×time interaction.

The 6-month follow-up data were analyzed separately for the intervention and control group (ie, only within-group changes analyzed) because the control group had gained access to the CDMI intervention by that time. Growth curves were examined to determine whether any effects in the intervention group remained or increased at 6 months (ie, no significant decrease in effect between T1 and T2) and whether effects increased in the control group after the 3-month follow-up (ie, significant increase in effect between T1 and T2). An exploratory analysis was undertaken into the relationship between intervention use (number of log-ins) and effectiveness within the intervention group during the 6-month intervention period. To this end, the interaction between use and time was tested in a linear mixed model.

All analyses were adjusted for baseline demographic factors (age, gender, living arrangement, and education level). Adjustments were also made for factors that were related to dropout as indicated by the attrition analysis. Standardized effect sizes (Cohen *d*) were calculated using the estimated (adjusted) mean differences of the outcomes from the linear mixed models divided by the pooled standard deviation of the outcomes at baseline. Analyses were performed using SPSS version 22 and Stata version 12.1 statistical software packages.

## Results

### Recruitment of Participants

**Figure 1:**
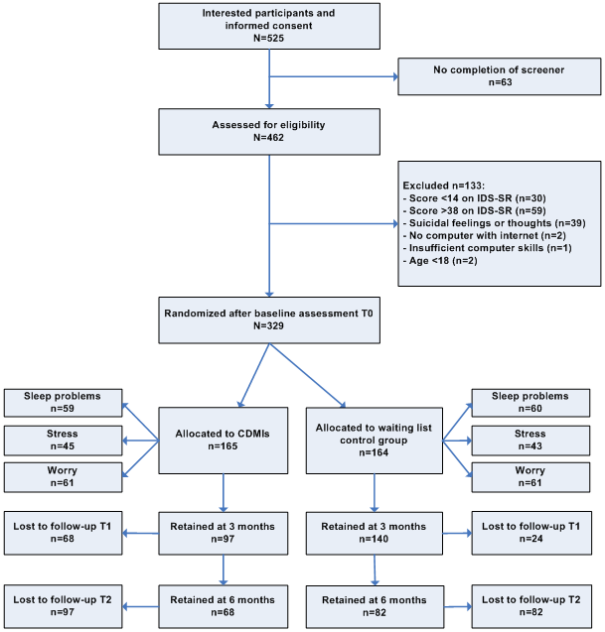
Flowchart of participants. CDMI: complaint-directed mini-intervention; IDS-SR: Inventory of Depressive Symptomatology Self-Report.

In total, 525 people showed interest in the study by giving their informed consent. These individuals were invited to complete the first (screening) part of the baseline questionnaire. Sixty-three people did not complete the baseline questionnaire and were excluded from the study. Another 133 people did not meet the inclusion criteria, primarily because of too few or too many depressive symptoms or because of the presence of suicidal thoughts or behaviors. This resulted in a study population of 329 participants, of which 165 were randomized to the experimental condition and 164 to the control condition. Of the 165 participants in the experimental condition, 59 participated in the sleep CDMI, 45 participated in the stress CDMI, and 61 participated in the worry CDMI. Figure 1 shows the flow of participants during the trial.

### Baseline Characteristics

Table 1 shows an overview of the baseline characteristics of the participants. The participants had a mean age of 43 (SD 12.93, range 18-81) years and the majority were female (75.7%, 249/329). More than 98% (323/329) had Dutch nationality, and a majority had a paid job (70.8%, 233/329), a high education level (72.0%, 237/329), and a high income (70.2%, 231/329). Participants in the control condition rated the experienced severity of their complaints as high less often (49.4%, 81/164) than the participants in the intervention condition (58.8%, 97/165).

**Table 1:** Baseline characteristics of the participants in the complaint-directed mini-intervention (CDMI) and control groups (N=329).

Characteristic		CDMI (n=165)	Control (n=164)
**Age (years)**			
	Mean (SD)	42.85 (12.83)	43.65 (13.05)
	Range	18-76	18-81
**Gender, n (%)**			
	Female	122 (73.9)	127 (77.4)
	Male	43 (26.1)	37 (22.6)
**Marital status, n (%)**			
	Single	83 (50.3)	84 (51.2)
	Living with partner	82 (49.7)	80 (48.8)
**Nationality, n (%)**			
	Dutch	2 (1.8)	4 (2.4)
	Other	163 (98.2)	160 (97.6)
**Living arrangement, n (%)**			
	Alone	40 (24.2)	39 (23.8)
	With others	125 (75.8)	125 (76.2)
**Education, n (%)**			
	Low	47 (27.4)	45 (28.5)
	High	118 (72.6)	119 (71.5)
**Income, n (%)**			
	Low	50 (30.3)	48 (29.3)
	High	115 (69.7)	116 (70.7)
**Employment, n (%)**			
	Paid	116 (70.3)	117 (71.3)
	No paid	49 (29.7)	47 (28.7)
**Duration complaints, n (%)**			
	<1 year	59 (35.8)	63 (38.4)
	≥1 year	106 (64.2)	101 (61.6)
**Severity complaints, n (%)**			
	Low	68 (41.2)	83 (50.6)
	High	97 (58.8)	81 (49.4)

### Attrition

At T1, data were available for 237 participants (dropout rate 28.0%, 92/329). The loss of participants at T1 was significantly higher in the experimental condition (41.2%, 68/165) compared to the control condition (14.6%, 24/164; χ^2^_1_=28.8, *P*<.001). Analysis of baseline factors showed there was a significant association between baseline anxiety scores and attrition. The mean score on the GAD-7 at T0 was 1.2 points lower in participants who completed the T1 assessment as compared to those who did not (*t*_327_=–2.45, *P*=.02). Consequently, we adjusted for this variable in all analyses. Ten participants indicated suicidal thoughts or plans at T1, of which eight belonged to the control condition and hence did not gain access to the CDMIs.

At T2, 150 participants completed the questionnaire (dropout rate 54.4%, 179/329). Again, attrition was higher in the experimental condition (58.8%, 97/165) than in the control condition (50.0%, 82/164), but this difference was not significant. Suicidal thoughts or plans were indicated by seven participants at T2: five from the experimental condition and two from the control condition.

### Effectiveness of the Intervention

The observed and estimated marginal means (estimated means adjusted for all factors in the model) for all outcomes at baseline and 3-month follow-up are presented in Table 2. The results of the linear mixed models analysis showed significant time×intervention effects for all outcomes except stress (see Table 3). This means that greater reductions in depression, sleep problems, worry, and anxiety were detected between baseline and the 3-month follow-up in the intervention group compared to the control group. Moreover, greater improvements in well-being were observed over time in the intervention group. The intervention did not have a significant effect on stress complaints. The standardized effect size for the primary outcome of depression was large (Cohen *d*=–0.70). The magnitude of the effect sizes for the secondary outcomes were generally in the small-to-medium range across the outcomes (Cohen *d*=–0.20 to 0.40).

**Table 2:** Observed and estimated marginal means (EMM) of the outcomes at baseline and 3-month follow-up.

Outcome		Observed means, mean (SD)		Estimated means, EMM (SEM^a^)	
		CDMI^b^ intervention (n=165)	Control (n=164)	CDMI intervention (n=165)	Control (n=164)
**Depression (IDS-SR^c^)**					
	Baseline	26.08 (6.53)	25.01 (6.16)	25.69 (0.64)	24.91 (0.65)
	3 months	20.34 (9.54)	24.28 (9.40)	20.35 (0.79)	24.04 (0.69)
**Sleep (JSEQ^d^)**					
	Baseline	11.61 (5.42)	11.21 (5.34)	11.98 (0.48)	11.78 (0.48)
	3 months	8.91 (5.25)	10.62 (5.41)	9.48 (0.55)	11.03 (0.50)
**Stress (PSS^e^)**					
	Baseline	21.82 (5.86)	21.48 (5.37)	21.53 (0.47)	21.35 (0.48)
	3 months	17.71 (6.54)	18.97 (5.73)	17.87 (0.57)	18.80 (0.50)
**Worry (PSWQ^f^)**					
	Baseline	37.76 (9.26)	38.28 (9.61)	36.92 (0.77)	37.61 (0.78)
	3 months	32.46 (10.07)	36.44 (9.70)	31.88 (0.88)	35.81 (0.81)
**Anxiety (GAD-7^g^)**					
	Baseline	10.09 (4.16)	10.04 (3.73)	10.24 (0.35)	10.42 (0.35)
	3 months	6.37 (4.27)	7.76 (4.06)	6.87 (0.42)	8.19 (0.37)
**Well-being (WEMWBS^h^)**					
	Baseline	42.99 (6.03)	43.66 (6.67)	42.84 (0.60)	43.25 (0.61)
	3 months	46.53 (7.73)	44.34 (7.38)	46.17 (0.73)	44.01 (0.64)

^a^SEM: standard error of the mean.

^b^CDMI: complaint-directed mini-intervention.

^c^IDS-SR: Inventory of Depressive Symptomatology Self-Report.

^d^JSEQ: Jenkins Sleep Evaluation Questionnaire.

^e^PSS: Perceived Stress Scale.

^f^PSWQ: Penn State Worry Questionnaire.

^g^GAD-7: Generalized Anxiety Disorder Scale.

^h^WEMWBS: Warwick-Edinburgh Mental Well-being Scale.

**Table 3:** Estimated differences in mean change of outcomes (crude and adjusted)^a^ between baseline and 3-month follow-up for the for complaint-directed mini-intervention (CDMI) intervention group versus the control group.^b^

Outcome		Estimate of mean change difference (95% CI)	*t* (*df*)	*P*	Cohen *d* (95% CI)
**Depression (IDS^c^)**					
	Crude model	–4.78 (–6.88 to –2.68)	–4.49 (297)	<.001	
	Adjusted model	–4.47 (–6.54 to –2.40)	–4.25 (309)	<.001	–0.70 (–1.03 to –0.38)
**Sleep (JSEQ^d^)**					
	Crude model	–1.79 (–2.93 to –0.65)	–3.10 (258)	.002	
	Adjusted model	–1.75 (–2.89 to –0.62)	–3.05 (262)	.003	–0.33 (–0.54 to –0.12)
**Stress (PSS^e^)**					
	Crude model	–1.32 (–2.77 to 0.13)	–1.79 (271)	.08	
	Adjusted model	–1.12 (–2.55 to 0.31)	–1.54 (284)	.12	–0.20 (–0.45 to 0.06)
**Worry (PSWQ^f^)**					
	Crude model	–3.56 (–5.37 to –1.76)	–3.89 (260)	<.001	
	Adjusted model	–3.25 (–5.04 to –1.47)	–3.59 (270)	<.001	–0.34 (–0.53 to –0.16)
**Anxiety (GAD-7^g^)**					
	Crude model	–1.17 (–2.22 to –0.12)	–2.19 (267)	.03	
	Adjusted model^h^	–1.14 (–2.19 to –0.09)	–2.13 (271)	.03	–0.29 (–0.55 to –0.02)
**Well-being (WEMWBS^i^)**					
	Crude model	2.74 (0.87 to 4.61)	2.89 (289)	.004	
	Adjusted model	2.57 (0.70 to 4.44)	2.70 (289)	.007	0.40 (0.11 to 0.70)

^a^Crude model: crude association (model includes only intervention condition, time, and time×intervention condition). Adjusted model: adjusted for age, gender, living arrangement, education, symptom severity, and anxiety scores at baseline.

^b^Regression coefficient for the time×condition interaction term.

^c^IDS-SR: Inventory of Depressive Symptomatology Self-Report.

^d^JSEQ: Jenkins Sleep Evaluation Questionnaire.

^e^PSS: Perceived Stress Scale.

^f^PSWQ: Penn State Worry Questionnaire.

^g^GAD-7: Generalized Anxiety Disorder Scale.

^h^Model 2 for anxiety does not include baseline anxiety scores as a covariate because they are included in the outcome.

^i^WEMWBS: Warwick-Edinburgh Mental Well-being Scale.

### Outcomes at 6-Month Follow-Up

**Figure 2:**
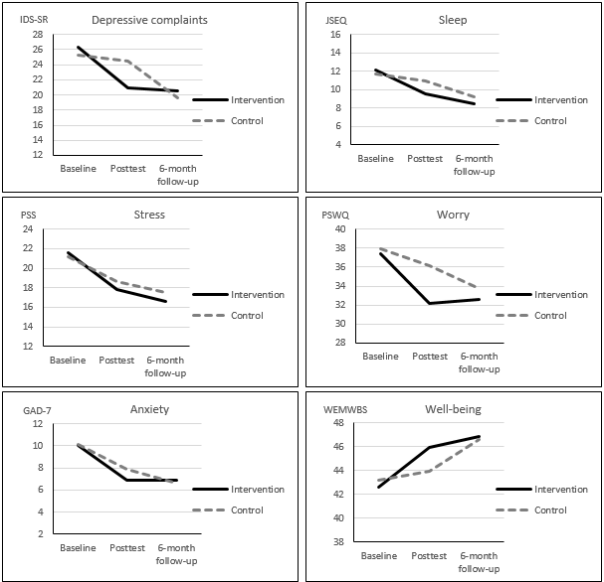
Estimated marginal means for outcomes in the intervention and control groups for total follow-up period.

Figure 2 shows the course of the (estimated) primary and secondary outcomes in the two groups. In the intervention group, the greatest effects were observed between baseline and T1. These effects did not significantly increase or decrease at the 6-month follow-up, except for further reductions in sleep complaints (see Table 4). Generally, effects in the intervention group were sustained until 6 months. This pattern was reversed in the control group because they did not receive the intervention until after the T1 assessment. The greatest effects were observed between T1 and T2 in the control group, and were of similar magnitude as those found between T0 and T1 in the intervention group (see Table 4). However, there were some exceptions. Stress complaints showed a similar pattern of change in both groups: the greatest reduction between T1 and T0 (which is also reflected in the nonsignificant difference in the effectiveness analysis, see previous), and a smaller reduction between T1 and T2. Moreover, anxiety complaints also seemed to decrease a little more between T0 and T1 than between T1 and T2 in the control group.

**Table 4:** Within-group estimated changes in outcomes between T1 and T0 and between T2 and T1.^a^

Outcome		*F* (*df*)		*P*		Intervention				Control			
						Estimate (95% CI)		*P*		Estimate (95% CI)		*P*	
**Depression**													
	Time		44.49 (2,482)		<.001								
	T1 vs T0						–5.40 (–7.00 to –3.81)		<.001		–0.84 (–2.24 to 0.56)		.24
	T2 vs T1						–0.41 (–2.37 to 1.55)		.68		–4.83 (–6.55 to –3.11)		<.001
**Sleep**													
	Time		42.40 (2,426)		<.001								
	T1 vs T0						–2.54 (–3.41 to –1.67)		<.001		–0.75 (–1.49 to 0.00)		.05
	T2 vs T1						–1.11 (–2.17 to –0.06)		.04		–1.74 (–2.67 to –0.81)		<.001
**Stress**													
	Time		67.14 (2,452)		<.001								
	T1 vs T0						–3.75 (–4.82 to –2.68)		<.001		–2.56 (–3.48 to –1.63)		<.001
	T2 vs T1						–1.21 (–2.51 to 0.09)		.07		–1.17 (–2.31 to –0.03)		.045
**Worry**													
	Time		43.99 (2,429)		<.001								
	T1 vs T0						–5.23 (–6.64 to –3.83)		<.001		–1.74 (–2.94 to –0.54)		.004
	T2 vs T1						0.46 (–1.25 to 2.17)		.60		–2.42 (–3.91 to –0.93)		.002
**Anxiety^b^**													
	Time		81.47 (2,440)		<.001								
	T1 vs T0						–3.41 (–4.20 to –2.61)		<.001		–2.22 (–2.92 to –1.53)		<.001
	T2 vs T1						0.19 (–0.78 to 1.16)		.70		–1.23 (–2.08 to –0.37)		.005
**Well-being**													
	Time		27.11 (2,462)		<.001								
	T1 vs T0						3.40 (2.03 to 4.76)		<.001		0.77 (–0.42 to 1.95)		.21
	T2 vs T1						0.84 (–0.82 to 2.5)		.32		2.63 (1.17 to 4.09)		<.001

^a^All estimates are adjusted for age, gender, living arrangement, education, symptom severity, and anxiety scores at baseline.

^b^Estimate for anxiety does not include baseline anxiety scores as a covariate because they were included in the outcome.

### Use of and Satisfaction With Intervention

Approximately three-quarters of the intervention group actually created an account (127/165, 77.0%); of these participants, 91.3% (116/127) logged into their chosen CDMI at least once. During the 3-month intervention period, the participants logged in a median 3 times (range 0-166, IQR=5). Approximately half of the control group actually created an account (86/164, 52.4%) after completing the T1 measurement; of these participants, 85% (73/86) logged into their chosen CDMI at least once. During the 3-month period between T1 and T2, the participants in the control group logged in a median 2 times (range 0-85, IQR=4). Of the 75 participants in the intervention group who completed the use and satisfaction questions, a majority (56%, 42/75) reported that they spent an average of 30 minutes or more a week on the CDMIs and 8% (6/75) of participants spent 2 hours or more a week on the CDMIs. Of the 48 participants in the control group who completed the use and satisfaction questions, a majority (54%, 26/48) reported that they spent an average of 30 minutes or more a week on the CDMIs and 10% (5/48) spent 2 hours or more a week on the CDMIs.

Exploratory analysis into the relationship between log-ins and effectiveness showed no discernible dose-response relationship, and this was underlined by the finding that there were no significant interaction effects between the number of log-ins and time (see estimated marginal means and test statistics in Multimedia Appendix 3). However, although the overall tests of the interaction terms across the complaints were not significant, there were a number of significant contrasts. Participants who logged in four or more times showed greater decreases in depressive complaints between baseline and 3-month follow-up than those who did not log in (*t*_226_=–2.12, *P*=.04). Those who logged in one to three times showed greater decreases in worry complaints between baseline and 3-month follow-up than those who did not log in (*t*_191_=–1.98, *P*=.049).

**Figure 3:**
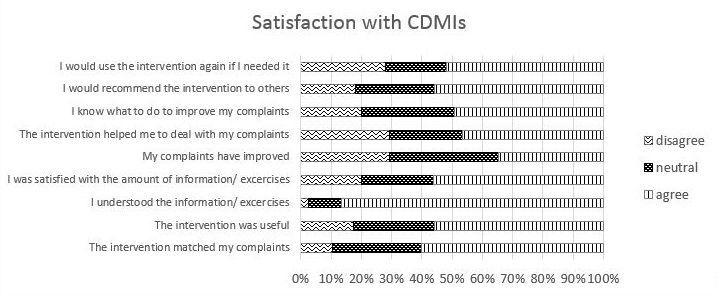
Satisfaction with complaint-directed mini-interventions (CDMIs).

Overall satisfaction with the intervention was moderate to high. The median satisfaction score given by the intervention group (n=75) was 7 (on a scale of 1 to 10) and the mean was 6.3 (SD 1.8). Participants in the control group who accessed the intervention gave a near identical satisfaction rating (n=45; mean 6.4, SD 1.5). Figure 3 shows the ratings participants in the intervention group scored on the various satisfaction items. These results show a similar pattern of moderate to high satisfaction across the various topics.

Various suggestions were made for future improvements. Among the most mentioned were to include reminders, additional information, and more structure to exercises.

## Discussion

### Summary of Findings

This RCT evaluated the effectiveness of unguided Web-based self-help interventions that aimed to decrease depressive complaints by targeting stress, sleep problems, or worry. The results at 3-month follow-up showed a significant reduction in depressive symptoms for participants in the intervention group compared to participants in the wait-list control group. Furthermore, significant effects were observed for sleep problems, worry, anxiety, and well-being. The CDMIs did not significantly reduce stress complaints, but reductions were seen in both the intervention and control groups. At 6-month follow-up, the improvements in the intervention group were generally sustained. The control group, which started with the CDMIs after 3 months, experienced significant improvements at the 6-month follow-up compared to the 3-month follow-up for all outcome variables but stress. In general, participants were moderately to highly satisfied with the CDMIs.

### Comparison With Other Studies

Online self-help interventions targeting symptoms of depression and the prevention of cases of depressive disorder have been found to be effective in previous studies [9,28,49]. A recent meta-analysis found that online interventions for the prevention of depression can reduce depressive symptoms in the short and medium term compared to no-intervention control conditions (eg, short-term pooled effect size of Hedges *g*=–0.35, 95% CI –0.57 to –0.12) [28]. A meta-analysis by Davies and colleagues [49] also showed that computer-delivered and Web-based interventions can be effective in improving depression in university students, with a pooled standardized mean difference of Hedges *g*=–0.43 (95% CI –0.63 to –0.22, *P*<.001). Zhou and colleagues [50] conducted a meta-analysis to the efficacy of Internet-based cognitive behavioral therapy (CBT) for people who fulfill the criteria for at least subthreshold depression. They concluded that in the short-term the efficacy of Internet-based CBT is superior to a nonactive control group (pooled standardized mean difference of –0.28, 95% CI −0.42 to −0.14), whereas the long-term efficacy is inconclusive. Moreover, a recently published meta-analysis about the effectiveness of online mindfulness-based interventions reported a small effect size on depressive symptoms (Hedges *g*=0.29, 95% CI 0.13-0.46, *P*=.001) [41].

The effect size for depressive symptoms found in our study (Cohen *d*=–0.70) is somewhat larger than reported in these meta-analyses. In some cases, this may be due to more restrictive eligibility criteria that were applied in these meta-analyses, including diagnosis and exclusion of depressive disorders at trial entry (eg, [28]), compared to our study. However, other online, unguided psychological interventions for participants with increased levels of depressive symptomatology also showed lower effect sizes [51].

### Methodological Considerations

First, we encountered a high dropout rate of participants during the course of the study. High dropout rates are a problem often encountered in eHealth trials [52,53], with rates as high as 50% to 72% reported. Dropout rates tend to be higher for unguided versus guided eHealth trials [53]. Dropout also tends to be higher in intervention groups than control groups as was the case in our study. Although this is not an uncommon occurrence in eHealth studies, it may nonetheless give rise to attrition bias. However, analyses showed that the only measured difference between participants who completed the T1 assessment versus those who did not was the mean score on the GAD-7 (anxiety), which was adjusted for in the analyses. Furthermore, in the analyses, all participants were included according to the intention-to-treat principle and missing data was accounted for by using linear mixed models (ie, maximum likelihood), which is a recommended strategy for estimating unbiased treatment effects [54].

Second, the CDMIs were developed as a preventive approach to depression, but the diagnostic status of the participants in this study was not assessed with a clinical interview because it was not feasible. To avoid being too restrictive and denying people access to the CDMIs who might benefit from the interventions, we included people with mild and moderate depressive symptoms in our trial. We cannot rule out that some participants in the trial might have fulfilled diagnostic criteria for depression at trial entry. Therefore, the findings and effect sizes should be interpreted in light of this fact.

Third, the participants could not be blinded. The reduction in complaints observed by the control group at T2 versus T1 were comparable to the reduction seen in the intervention group at T1 versus T0. Therefore, it is likely that the effects found are not largely influenced by the lack of blinding.

Another point of consideration is the limited use of the intervention, despite the positive effects. The use of the CDMIs was slightly more limited in the control group. This might have been due to a reduction in complaints in the control condition in the first 3 months of the study, reducing the need for the control condition to use the CDMIs once access was provided. The evaluation of the CDMIs by the users does suggest there is room for improvement of the intervention. Adding reminders was a frequently given suggestion for improvement. However, it would be worthwhile to further question users about strategies that may increase the use of the intervention. We conducted some exploratory analyses to examine the relationship between the number of log-ins and effectiveness, which showed no discernible dose-response relationship. However, it is important to interpret these exploratory findings with caution due to potential selection bias. Moreover, different operationalizations of adherence may be differentially related to effectiveness in eHealth research [55], which would be interesting to examine in future research.

Fifth, although our trial applied an open recruitment strategy, meaning that there were no restrictions for participation other than the inclusion criteria, it is possible that potential participants were missed due to the use of mainly Internet-based recruitment avenues. On the other hand, people who do not use the Internet might be less open to using an unguided Web-based self-help intervention. Nevertheless, the participants of the trial might not have truly reflected the general Dutch adult population with access to the Internet and sufficient computer skills.

Finally, the study population consisted of mainly highly educated female participants. Therefore, the generalizability of the findings with respect to males and other educational levels remains to be determined. Moreover, as mentioned in the Introduction, the CDMIs were developed to also suit a range of target groups, including the low SES population, but participation in the CDMIs and in this study was not restricted to individuals with a low SES. The baseline characteristics indicate that 72% of the participants had a high educational level. This means that without specific efforts aimed at inclusion of low SES people, the usage of the intervention among this group will be relatively low.

### Future Research Directions

Integration of the CDMIs in primary care may be a useful next step for several reasons. As mentioned in the Introduction, patients frequently present to their GP with psychological stress, sleep problems, and worry [17-21]. Moreover, the guidelines of the Dutch College of General Practitioners recommend e-mental health as first step interventions for patients with depressive complaints. During and after the trial, we received several requests from GPs and GP nurses for information about and the use of the CDMIs. As such, the CDMIs seem to be suitable interventions for use in the GP setting that satisfy a demand. When implementing the interventions in the GP setting, the CDMIs can also be offered with some guidance from the GP nurse, which might be a way of boosting adherence and effectiveness [30,31]. Guidance may also be needed to increase the reach to low SES populations.

In addition to human guidance, technology itself may offer opportunities for support through persuasive system design features [56], which may be just as effective as human support [57]. Prompts are an example of a feature that may increase adherence [58]. Based on user feedback, we have recently developed an app to supplement the CDMIs by providing motivational quotes, tips, and prompts to use the CDMIs. Recent advances in mHealth provide opportunities for systems to learn about their user and offer meaningful personalized interventions when they are necessary and, thus, potentially offering more sophisticated technology-based support with or without human support (eg, [59]). It is likely that such personalized interventions will contribute to adherence and ultimately to effectiveness, and it would be interesting to explore this avenue more in future research.

A focus on research questions addressing the use and mechanisms of change in online interventions would be a fruitful next step. This is also in line with calls to pay more attention to the evaluation of intervention components rather than intervention packages in alternative research designs to traditional RCTs as a means to better tie in with the rapid and flexible nature of e-mental health development [60,61]. With respect to the CDMIs, as noted by one of the anonymous reviewers of this paper, it may be worthwhile in the future to investigate the effect of user choice. To this end, a comparison could be made between persons who are able to choose their modules and exercises versus those who are restricted to a more traditional approach in which participants have to go through modules sequentially. Also gaining more insight into the optimal amount of time, exercises, or modules that are needed, and the relationship with user characteristics would be valuable to better personalize interventions in the future. Currently, participants of the CDMIs are advised to spend 2 to 3 hours a week for a period of at least 4 weeks. However, a recent study of Bunge et al [62] showed that even a brief, unsupported Internet intervention of 5 to 10 minutes improved depression scores at 1-week follow-up. Finally, future research should focus on gaining insights into the most optimal strategies for reaching and engaging low SES populations in (preventive) interventions targeting psychological complaints, such as the CDMIs.

In conclusion, the online CDMIs were successful in significantly reducing depressive complaints and had an effect on, albeit to a lesser extent, sleep problems, worry, anxiety, and well-being. The intervention did not significantly reduce stress complaints. This study shows that a purely online self-help intervention can have a positive impact on mental health outcomes. Future research should focus on which specific strategies may boost adherence, and increase the reach of the CDMIs among people with low SES.

## Appendix

Multimedia Appendix 1 CONSORT-EHEALTH checklist (V1.6.1).

Multimedia Appendix 2 Additional information and complaint-directed mini-intervention (CDMI) screenshots.

Multimedia Appendix 3 Intervention use (number of logins) and effect sizes.

## Abbreviations

CBT: cognitive behavioral therapy
CDMI: complaint-directed mini-intervention
EMM: estimated marginal means
GAD-7: Generalized Anxiety Disorder Scale
GP: general practitioner
IDS-SR: Inventory of Depressive Symptomatology Self-Report
JSEQ: Jenkins Sleep Evaluation Questionnaire
NTR: Netherlands Trial Register
RCT: randomized controlled trial
PSS: Perceived Stress Scale
PSWQ: Penn State Worry Questionnaire
SES: socioeconomic status
WEMWBS: Warwick-Edinburgh Mental Well-being Scale
