# Supplementary material for: Complaint-Directed Mini-Interventions for Depressive Complaints: A Randomized Controlled Trial of Unguided Web-Based Self-Help Interventions
Source: J Med Internet Res. 2017 Jan 4;19(1):e4. doi: 10.2196/jmir.6581 (PMC5244033; doi:10.2196/jmir.6581)
Supplement: Multimedia Appendix 3 [file jmir_v19i1e4_app3.pdf]

	0 logins[a] (n=49)	1-3 logins (N=57)	4 or more logins (N=59)	Test Time*login group	
	EMM (SEM)[b]	EMM (SEM)	EMM (SEM)	F_{df};	p-value
**Depression (IDS)**				$1.45_{4, 220}$	0.22
Baseline	25.51 (1.19)	25.71 (1.13)	26.89 (1.07)		
3 months	22.96 (1.68)	19.79 (1.41)	19.72 (1.23)		
6 months	19.33 (1.98)	20.95 (1.65)	20.09 (1.41)		
**Sleep (JSEQ)**				$0.27_{4, 188}$	0.90
Baseline	11.96 (0.76)	11.27 (0.72)	11.62 (0.68)		
3-months	10.11 (1.03)	8.16 (0.88)	9.18 (0.77)		
6 months	8.71 (1.19)	7.63 (1.00)	7.90 (0.87)		
**Stress (PSS)**				$0.83_{4, 198}$	0.51
Baseline	20.98 (0.88)	21.89 (0.83)	22.43 (0.79)		
3-months	17.31 (1.25)	17.68 (1.03)	18.41 (0.90)		
6 months	17.77 (1.44)	16.91 (1.20)	16.13 (1.03)		
**Worry (PSWQ)**				$2.23_{2,184}$	0.07
Baseline	35.33 (1.36)	39.51 (1.28)	38.13 (1.22)		
3-months	31.84 (1.75)	32.16 (1.48)	32.84 (1.33)		
6 months	31.05 (1.94)	36.06 (1.66)	31.73 (1.47)		
**Anxiety (GAD-7)**				$1.11_{4,193}$	0.40
Baseline	9.41 (0.59)	11.02 (0.56)	9.80 (0.53)		
3 months	7.20 (0.84)	6.87 (0.70)	6.21 (0.61)		
6 months	7.37 (0.99)	6.76 (0.82)	6.54 (0.70)		
**Wellbeing (WEMWBS)**				$0.58_{4, 214}$	0.68
Baseline	43.14 (0.97)	43.20 (0.92)	42.69 (0.87)		
3-months	46.00 (1.39)	47.21 (1.16)	46.25 (1.01)		
6 months	47.70 (1.64)	46.20 (1.37)	47.89 (1.15)		
